# Varix of the vortex vein ampulla: a case report and imaging correlation

**DOI:** 10.3389/fmed.2025.1685952

**Published:** 2025-10-21

**Authors:** Tong-tong Niu, Yun Xiao

**Affiliations:** Department of Ophthalmology, Xinjiang 474 Hospital, Ürümqi, China

**Keywords:** vortex vein, varix of vortex vein ampulla, choroid, ICGA, pressure

## Abstract

**Background:**

We report a case of varix of the vortex vein ampulla (VVVA) in the right eye. VVVA is relatively rare and often presents as a fundus mass-like lesion during clinical examination, making it easily confused with choroidal tumors. This case report aims to improve ophthalmologists’ understanding of this rare lesion and provide a reference for clinical diagnosis and management.

**Case presentation:**

A 29-year-old male presented with 1 month of decreased vision in the right eye. His best-corrected visual acuity was 20/20. B-scan ultrasonography and optical coherence tomography (OCT) revealed a choroidal space-occupying lesion. Fundus examination identified a deep red elevated mass at the inferotemporal VVVA of the right eye. Further indocyanine green (ICG) angiography confirmed VVVA. The mass became more prominent when the patient gazed downward with the right eye. After applying pressure to the eye, follow-up B-scan and OCT showed the mass had disappeared or reduced—findings that strongly confirmed the lesion was VVVA rather than a choroidal tumor.

**Conclusion:**

VVVA is an uncommon condition. This case reminds ophthalmologists to consider less common causes when evaluating choroidal mass-like lesions. Accurate diagnosis of VVVA is critical to avoid misdiagnosis as a choroidal tumor and unnecessary invasive testing or treatment. Recognition of this benign condition can prevent misdiagnosis as a choroidal tumor and unnecessary invasive testing.

## Background

The choroid, as a vital structure within the eye, is situated between the retina and the sclera. It presents as a soft, smooth, elastic, and vascularly rich brown membrane. The vortex veins serve as the sole venous drainage system for the choroid, primarily responsible for draining blood from the choroidal, ciliary body, and iris vasculature. Additionally, they participate in the drainage of blood from some of the scleral vascular plexuses and the limbal vascular network. Hemodynamically, the veins in the posterior choroid tend to converge anteriorly, while those anterior to the equator converge posteriorly, ultimately forming the vortex venous structure through the progressive fusion of multiple venous levels. Notably, in the critical regions of venous convergence, localized enlargements of the venous lumen are frequently observed, giving rise to the characteristic ampullary morphology (1). However, this structure exhibits variability among individuals, with the vortex venous ampullae being inconspicuous in some populations.

In some patients, the vortex venous ampullae may undergo dilation, manifesting in the eye as brownish-red or black elevated masses (2). Choroidal elevations can be visualized on both ocular B-scan ultrasonography and optical coherence tomography (OCT), often prompting a series of investigations as if they were choroidal tumors (3). Therefore, a thorough understanding of the pathophysiological mechanisms and clinical manifestations of vortex venous ampullary dilation is of paramount importance for guiding precise clinical diagnosis and avoiding unnecessary medical investigations. We report a case involving a young male patient who presented with blurred vision in his right eye and requested an eye examination for spectacle prescription change. During the examination, a brownish-red elevation in the inferotemporal choroid of the right eye was detected. Following ocular B-scan ultrasonography, OCT, and indocyanine green (ICG) angiography, a diagnosis of right eye vortex venous ampullary dilation was ultimately established. The case is now reported as follows.

## Case presentation

**Figure 1:**
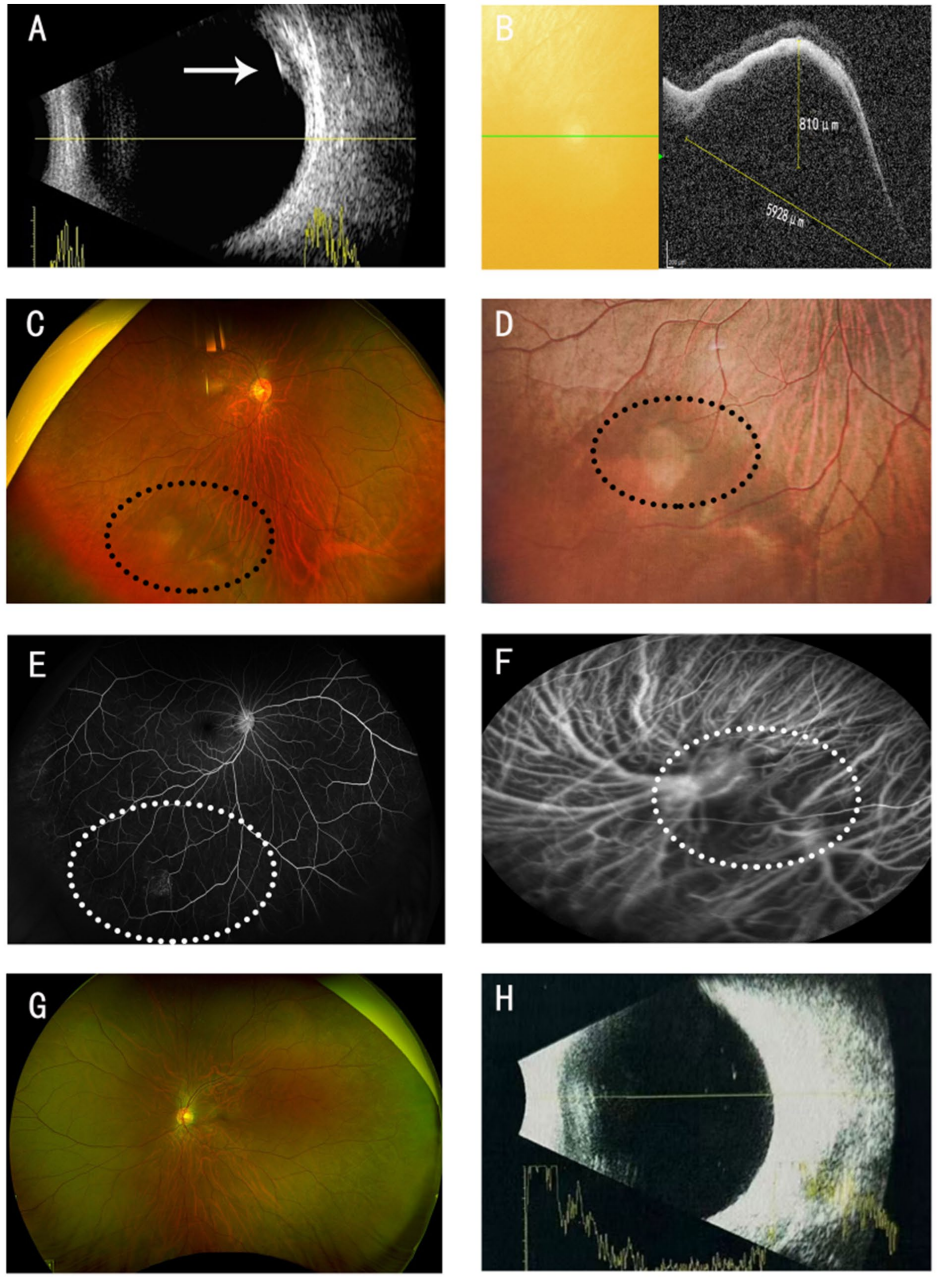
Right eye: **(A)** B-scan ultrasonography of the eye: Choroidal space-occupying lesion with a base diameter of 7.51 mm and a height of 2.51 mm, as indicated by the white arrow. **(B)** OCT: A low-reflective cavity with a height of 810 μm and a basal diameter of 5,928 μm is visible. **(C,D)** Fundus photography: A brownish-red elevated mass is visible in the inferotemporal region, as indicated by the black circle. **(E)** FFA: Localized fluorescein leakage is observed in the inferotemporal lesion, resulting in hyperfluorescence in the lesion area, as indicated by the white circle. **(F)** Dilation of the vortex vein ampulla, as indicated by the white circle. **(G,H)** Fundus photography, B-ultrasound: no abnormalities.

A 29-year-old male presented with a 1-month history of blurred vision in the right eye. Visual acuity was 20/40 in the right eye and 30/40 in the left eye. The patient presented for a routine refraction; with corrective lenses [−5.75 diopters (D) in the right eye and −5.25 D in the left eye], his best-corrected visual acuity improved to 20/20 in both eyes. Intraocular pressure was 15 mmHg in the right eye and 16 mmHg in the left eye. No abnormalities were observed in the anterior segments of either eye. B-scan ultrasonography revealed a solid echo in the inferotemporal region of the right eye, with heterogeneous internal echoes and relatively distinct borders, suggestive of a choroidal origin (Figure 1A). Optical coherence tomography (OCT) showed a choroidal elevation in the inferotemporal region of the right eye, measuring approximately 810 μm in height and 5,928 μm in basal diameter (Figure 1B). A reddish-brown choroidal elevation was observed in the inferotemporal retina of the right eye (Figures 1C,D). Fluorescein fundus angiography (FFA) showed no abnormalities during the arterial or venous phases; however, in the late phase, localized fluorescein leakage was noted in the inferotemporal lesion of the right eye, resulting in hyperfluorescence in the lesion area (Figure 1E). Indocyanine green angiography (ICGA) demonstrated dilation of the vortex vein ampulla in the inferotemporal region of the right eye (Figure 1F). The left eye fundus was unremarkable (Figures 1G,H).

**Figure 2:**
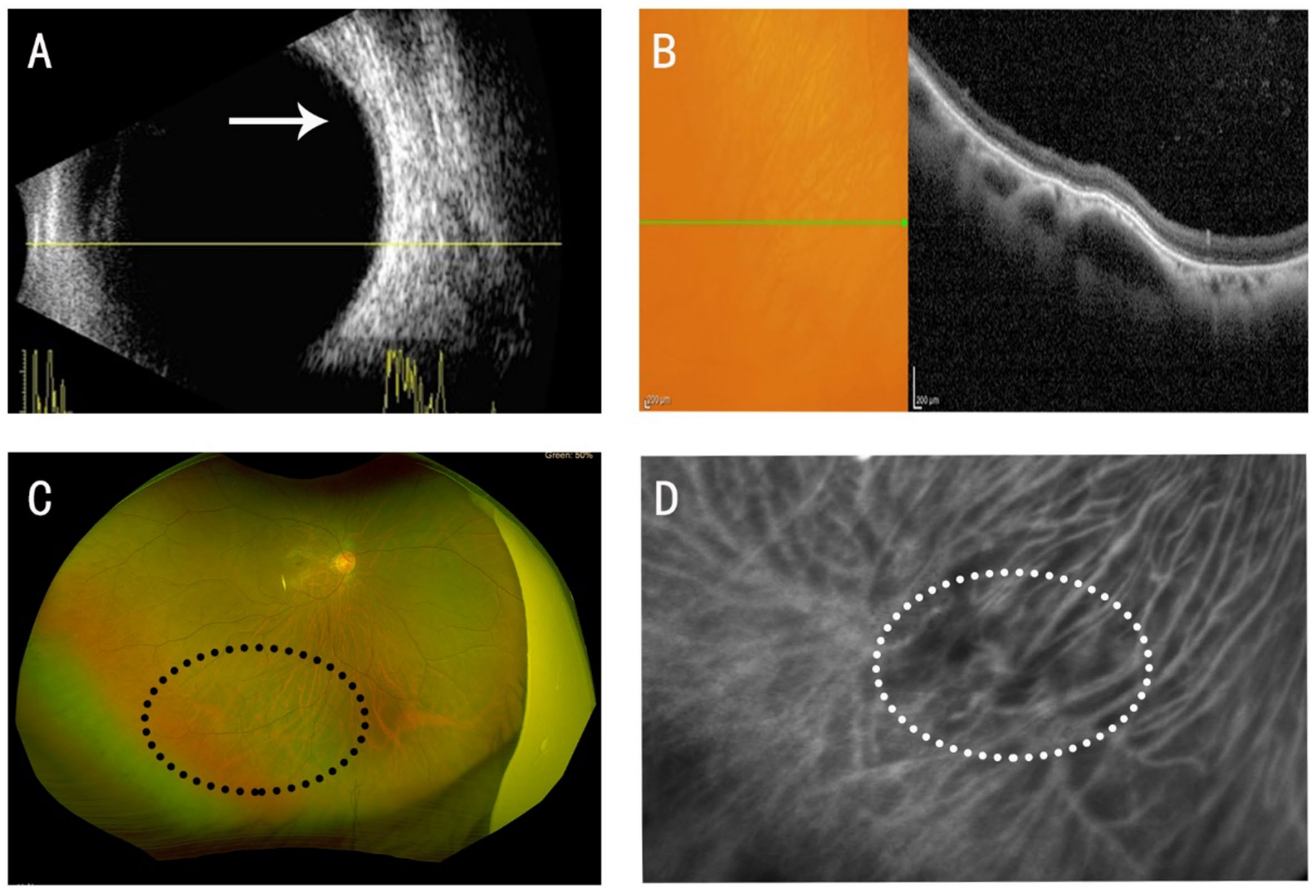
After applying appropriate pressure using an ophthalmic B-scan ultrasound probe to the patient’s eyeball. **(A)** B-scan ultrasonography: Resolution of the choroidal space-occupying lesion, as indicated by the white arrow. **(B)** OCT: Significant reduction in the retinal low-reflective cavity. **(C)** Fundus photography: Disappearance of the brownish-red mass in the inferotemporal region, as indicated by the black circle. **(D)** Near-infrared imaging: Reduced dilation of the vortex vein ampulla, as indicated by the white circle.

Two weeks later, we conducted a follow-up examination on the patient. After applying appropriate pressure to the patient’s eyeball using an ophthalmic B-scan ultrasound probe, B-scan ultrasonography showed resolution of the choroidal mass lesion in the right eye (Figure 2A). OCT revealed a significant reduction in the inferotemporal elevation of the right eye, with only a mild hyporeflective choroidal lesion remaining (Figure 2B). Fundus photography confirmed the disappearance of the inferotemporal elevated mass in the right eye (Figure 2C). Near-infrared photography showed reduced dilation of the vortex vein ampulla in the inferotemporal region of the right eye (Figure 2D).

## Discussion

Veins are crucial vascular structures in the eye, with relatively thin walls that possess a certain degree of elasticity, enabling them to adapt to pressure changes in blood flow. Their diameter is appropriately sized to ensure efficient blood return. In some ocular diseases, vortex veins may be affected. For instance, when inflammation, tumors, or vascular lesions occur in the eye, abnormalities in vortex vein blood flow may arise, such as alterations in blood flow velocity or vascular occlusion.

Previous reports have also documented vortex vein abnormalities (4). These patients presented with unilateral choroidal space-occupying lesions, often accompanied by symptoms such as ocular injection and pain, and some also experienced decreased visual acuity. However, these symptoms typically resolved spontaneously within a few weeks. Further fundus examination revealed involvement of the subchoroidal space with exudation, which led to the diagnosis of acute vortex vein occlusion. Although these patients also presented with vortex vein-related disorders, they differed significantly from the case reported in our study. Our patient had no ocular discomfort whatsoever, and the inferotemporal choroidal space-occupying lesion was incidentally detected during a routine ophthalmic examination. Additionally, there were no signs of hemorrhage or exudation in the fundus. Optical coherence tomography (OCT) only showed an elevated and expanded lesion with a hyporeflective appearance, and no other abnormal changes were observed. Notably, unlike prior studies (3, 5) which only documented the basic imaging features of vortex vein varix, our study systematically recorded real-time imaging changes before and after applying appropriate external pressure to the patient’s eyeball using an ophthalmic B-scan ultrasound probe. After pressure application, B-scan ultrasonography showed the disappearance of the space-occupying lesion, and the hyporeflective cavity on OCT also decreased significantly. These findings have not been fully covered in existing literature and suggest that our patient likely had only a physiological dilation of the vortex vein ampulla.

The etiology of varix of the vortex vein ampulla remains unclear. This is because its incidence is low, and some cases are misdiagnosed as choroidal tumors, choroidal nevi, or other conditions. Notably, several literature reports (5) show that the ampulla dilation becomes more obvious when patients gaze at the lesion site. We also observed this in our case: B-scan ultrasonography showed a higher elevation of the lesion when the patient gazed inferotemporally. The possible pathogenesis is that turning the eyeball toward the lesion increases pressure in the vortex vein, which enlarges the ampulla and causes corresponding fundus changes (5, 6). This also suggests that gaze-evoked kinking of the extrascleral vortex vein or narrowing of the scleral canal may be potential causes (7). Currently, varix of the vortex vein ampulla has been found in both myopic and hyperopic patients. So the role of scleral thickness and choroidal thickness in this disease is still unclear. We found that ampulla dilation disappears when pressure is applied to the eyeball. This happens mainly because the pressure affects ocular blood circulation: external force acts on the eyeball surface and squeezes intraocular blood vessels. This squeezing improves blood flow in the vortex vein. When blood return returns to normal, the increased pressure in the ampulla (caused by blood stasis) eases, and the dilated ampulla gradually returns to normal.

Previous studies have suggested that ultra-widefield indocyanine green angiography (ICGA) is helpful in identifying varix of vortex vein ampulla (2). In our case, we also observed dilated vortex veins on ICGA. However, due to the invasive nature of ICGA, the patient refused to undergo the procedure again during the follow-up. Instead, we opted for near-infrared fundus photography, which revealed a reduction in the size of the dilated vortex vein ampulla following pressure application to the eyeball, indicating a decrease in the dark area on the imaging. This strongly suggests that the mass in our patient’s eye was due to varix of vortex vein ampulla, rather than a tumor.

Varix of vortex vein ampulla does not affect visual acuity. In our patient, visual acuity improved to 20/20 in both eyes after a new prescription for glasses. However, when confronted with indications of choroidal space-occupying changes in clinical practice, a series of choroidal mass diseases, such as choroidal melanoma and choroidal metastatic carcinoma, are often considered. Enhancing our understanding of varix of vortex vein ampulla can help reduce unnecessary examinations and alleviate patient anxiety.

In summary, varix of vortex vein ampulla is a rare physiological change in the vortex veins that does not require treatment. The disappearance of the fundus mass upon external force application to the eye is a characteristic distinguishing feature.

## Data Availability

The raw data supporting the conclusions of this article will be made available by the authors, without undue reservation.
